# Primary transcatheter dilation of the pulmonary valve in cyanotic patients with tetralogy of Fallot and dominant pulmonary valve stenosis

**DOI:** 10.3389/fcvm.2024.1489413

**Published:** 2024-11-14

**Authors:** Nathalie Mini, Claudia Arenz, Marian Mikus, Martin B. E. Schneider

**Affiliations:** ^1^ Cardiac Catheterization Laboratories, Department of Pediatric Cardiology, German Pediatric Heart Centre, University Hospital Bonn, Bonn, Germany; ^2^ Department of Pediatric Cardiac Surgery, German Pediatric Heart Centre, University Hospital Bonn, Bonn, Germany; ^3^ Department of Anaesthesiology and Intensive Care Medicine, University Hospital Bonn, Bonn, Germany

**Keywords:** tetralogy of Fallot, pulmonary stenosis, pulmonary valve annulus, balloon dilation, cyanosis, spells

## Abstract

**Objectives:**

This study reviews the outcome of pulmonary valve dilation (PVB) in patients with tetralogy of Fallot (TOF) and predominantly pulmonary valve stenosis as first palliation and the impact of balloon-related cusp tears (BRCTs) on the surgical strategy.

**Background:**

The early management of cyanotic patients TOF is still controversial.

**Methods:**

This was a retrospective study of 19 patients with TOF who underwent PVB over 4 years. Differential growth of the pulmonary valve/annulus (PV) and arteries was documented, as was differential saturation improvement. Surgical findings were analyzed, including BRCT and subsequent surgical methods.

**Results:**

The median saturation value improved significantly from 70% (45%–98%) to 90% (74%–98%) (*p*-value = 0.03). Recurrent desaturation 7–45 days after the intervention occurred in 7 patients; 2 needed reinterventions, and 5 needed an early repair. At the time of repair, the median PV z-score improved from −3.7 (−6.12 to −1.3) to −2.1 (−4.2 to −0.19) (*p*-value = 0.2). The LPA z-score improved from −1.95 (−3.4 to −0.4) to 0.36 (−2.9 to 1.8) (*p*-value = 0.2), and the RPA z-score improved from −2 (−2.8 to 0.04) to 0.18 (−2.4 to 1.3) (*p*-value = 0.34). The mean pressure gradient decreased from 50 mmHg (32–72) to 38 mmHg (20–55) (*p*-value 0.08). The surgical repair was on time in 13 patients; one was still waiting for surgery. BRCTs were found in 8 patients and had no impact on the surgical strategy. Seven patients needed transannular patching, and in 11, the PV could be preserved (including 7 with BRCTs).

**Conclusion:**

Palliative transcatheter dilation of predominantly pulmonary valve stenosis in patients with TOF and predominantly pulmonary valve stenosis is safe and effective in palliating cyanosis in most patients. It can improve saturation and prompt pulmonary development, delaying the surgical repair to the right time. A subsequent BRCT seems to have no negative impact on the surgical strategy.

## Introduction

Tetralogy of Fallot (TOF) is the most common cyanotic congenital heart disease. Although surgical techniques have advanced significantly in recent years and early repair of TOF has become superior to transcatheter interventions as an approach to symptomatic infants in some centers, the early management of symptomatic patients with TOF is still controversial. For many reasons, some patients are still unfit for surgical repair and in need of alternative palliative strategies (like surgical creation of pulmonary aortic shunts, transcatheter dilation of the pulmonary stenosis, and stenting of the right outflow tract) to improve pulmonary flow and saturation.

A few studies have shown that transcatheter balloon dilation of the pulmonary valve (PV) could be effective in some patients with dominant pulmonary valve stenosis with no significant supravalvular or subvalvular pulmonary stenosis (1–3). Balloon dilation-related complications like cardiac tamponade, severe aortic regurgitation, infection, seizures, and transient pulmonary edema have been documented (4–6).

This study aims to review the results and outcomes of transcatheter balloon dilation of the PV in patients with Tof and predominantly pulmonary valve stenosis done in our center in the last four years and investigate the balloon valvulotomy's impact on the valve's surgical repair.

## Patients and methods

Between January 2020 and March 2024, we retrospectively recruited 19 patients with tetralogy of Fallot (TOF) and significant pulmonary stenosis (PS) who required palliative balloon dilation of the stenosed pulmonary valve. This intervention addressed desaturation and relieved cyanosis as an initial palliative measure. The dominant pulmonary stenosis in the patients included in this study was at the level of the pulmonary valve/annulus.

Echocardiographic findings were analyzed before and after the intervention, including the size of the pulmonary annulus, the development of the pulmonary arteries, the associated subvalvular or supravalvular pulmonary stenosis, and the pressure gradient of the stenosis. The balloon size, the saturation improvement, and the intervention-related complications were documented. The surgical findings, including the balloon-related valve tear and the surgical strategy of the valve, were analyzed (Table 1). The intervention associated morbidities were summarized (Table 2).

**Table 1:** The size and morphology of the pulmonary valve at the time of intervention and intraoperatively.

*N*	Weight (kg)	PV (mm)	PV Z-score	Balloon (mm)	Redo	Time between BPV & operation (days)	Weight at operation (kg)	PVM	PV Zscore at operation	BRCI	Surgery	PR/PS after the operatoin
1	4.3	6		8		280		Tricuspid			Planned	
2	2.8	7		9	Early repair	12	3	Tricuspid		Non-facing leaflet (mild)	PVR (commissurotomy)	No
3	3.3	5	−3.7	8		150	6.7	Bicuspid	−1.1	Anterior leaflet (moderate)	TAP (Monocusp)	Severe PR (Normal RV size)
4	3.9	7	−3.4	10	Early repair	15	4	Tricuspid		Left leaflet (mild to moderate)	PVR (commissurotomy)	Severe PR (Normal RV size)
5	3.3	7	−1.5	10		130	6	Bicuspid	−0.5	nd	TAP (Monocusp)	No
6	3.1	4		7	Early repair	20	3.3	Bicuspid		nd	TAP (Monocusp)	No
7	1.8	5	−2.5	6 & 8	Redilation	210	6.4	Bicuspid	−0.2	Both leaflets (moderate)	PVR (Delamination plasty)	Mild PR Mild PS
8	3.8	4	−4.8	6		140	6	Bicuspid	−2.7	nd	PVR	No
9	3.8	7	−2.5	9		230	10.6	Tricuspid	−2.1	nd	PVR (commissurotomy)	Mild PS
10	3.2	5	−2.8	6		170	7.3	Bicuspid	−2.1	Anterior commissure (mild)	PVR (commissurotomy/valvotomy)	No
11	2.5	5	−2.1	7		180	5.8	Bicuspid	−0.2	nd	PVR (commissurotomy & delamination plasty)	No
12	5.3	9		12	Early repair	30	5.5	Tricuspid		nd	PVR (commissurotomy)	No
13	3.8	7	−1.3	8 &10	Redilation	100	6.5	Tricuspid	−0.4	nd	TAP (Monocusp)	Moderate PR (Normal RV size)
14	3.4	5	−3.8	7	Early repair	45	4.7	Bicuspid	−3.2	nd	TAP (Monocusp)	Moderate PR (Normal RV size)
15	2.8	4	−4.5	6		180	7.8	Bicuspid	−4.2	nd	TAP (Monocusp)	Moderate PR
16	2	3	−6.2	6		105	6.3	Bicuspid	−3.7	Anterior leaflet (moderate)	PVR (delamination plasty)	No
17	2.2	5	−3.7	8		120	4	Bicuspid	−2.6	nd	TAP (Monocusp)	Severe PR (Normal RV size)
18	3.3	5	−4.8	7		130	6.8	Bicuspid	−1.6	Both leaflets (mild)	PVR (commissurotomy & shaving))	Severe PR (Normal RV size)
19	6	5	−4	7		150	8.2	Tricuspid	−1.6	Anterior leaflet (mild to moderate)	PVR (commissurotomy/valvotomy)	NO

PV, pulmonary valve; PVM, pulmonary valve morphology; BRCT, balloon-related cusp tear; PVR, pulmonary valve reconstruction; TAP, transannular patching; nd, not documented; PR, pulmonary regurgitation.

**Table 2:** Demonstrates the associated morbidities at the intervention time.

Patient	Syndrome/prematurity/associatedproblems
1	–
2	DiGeorge + low birth weight
3	DiGeorge
4	Trisomy 21
5	DiGeorge
6	Prematurity
7	Prematurity (GA: 30 weeks)
8	Trisomy 21 + Prematurity (GA: 32 weeks)
9	–
10	Low birth weight
11	Prematurity (GA: 34 weeks)
12	Robin sequence syndrome with multiple morbidities
13	Epilepsy
14	Low birth weight
15	–
16	Prematurity (GA: 31 weeks)
17	Prematurity + abdominal problems (GA: 33 weeks)
18	Prematurity and its related problems (GA: 35 weeks)
19	Hepatopathy, portal vein thrombosis + abdominal problems

The size of the pulmonary arteries and the pulmonary valve (and the related z score) was documented at the intervention day and the day before the operation.

All patients were on a B-blocker (propranolol, 2-6 mg/Kg/d) at the time of intervention.

The indication of transcatheter palliation in all patients was set together with the surgical team.

## Statistical analysis

All statistical analyses were performed using SPSS version 22. Continuous variables were reported as median ± IQR and categorical variables as count (percentage). A paired *t*-test was applied to compare the means of pulmonary parameters, saturation, and pressure gradient before and after dilation. A chi-square test was used to compare categorical variables.

## Ethical statement

According to the decision of the local ethical committee (with running number 2024-353-BO), informed consent and patient agreement were waived due to the retrospective design of the study.

## Intervention description

All interventions were performed under sedation by an experienced anesthesiologist. The intervention was done via the jugular vein in 2 patients and the femoral vein in the rest. After introducing a 4-French (F) sheath (Terumo, Radiofocus®, Introducer II), an end-hole angiographic catheter (usually Terumo®, Radiofocus®, Glidecath™) was advanced into the right ventricle. A right ventricular angiography by hand (Cranial 30°) demonstrated the right ventricular outflow tract (RVOT), the pulmonary valve (PV), and the pulmonary arteries. The end-systolic size of the pulmonary annulus was then measured in the lateral view. Using a 0.014-inch coronary guidewire (WIZDOM™ Steerable Guidewire, or Mailmain™ Gide Wire), the valve was crossed and positioned in the lower lobe of the branch pulmonary artery. The low-pressure balloon (Tyshak®PTV balloon) size was chosen to be 2 mm more than the pulmonary ring size (balloon/valve ration 1.2-1.4). The median value of the balloon to pulmonary anulus ratio was 1.4 (1.3-2). In some patients, the lack of a balloon waist during the first intervention (due to the elasticity of the loop or underestimation of the valve diameter) led to using a second balloon with a diameter of 1 or 2 mm more than the first to obtain adequate dilation. However, the ratio was estimated based on the diameter of the last balloon used and the initial measurement of the ring diameter. with more than one balloon due to After the valve was dilated, a new RV cine angiogram was obtained, and the intervention's results were ensured via echocardiography and saturation improvement. The sheath was then removed, and hemostasis was performed.

## Results

**Figure 1:**
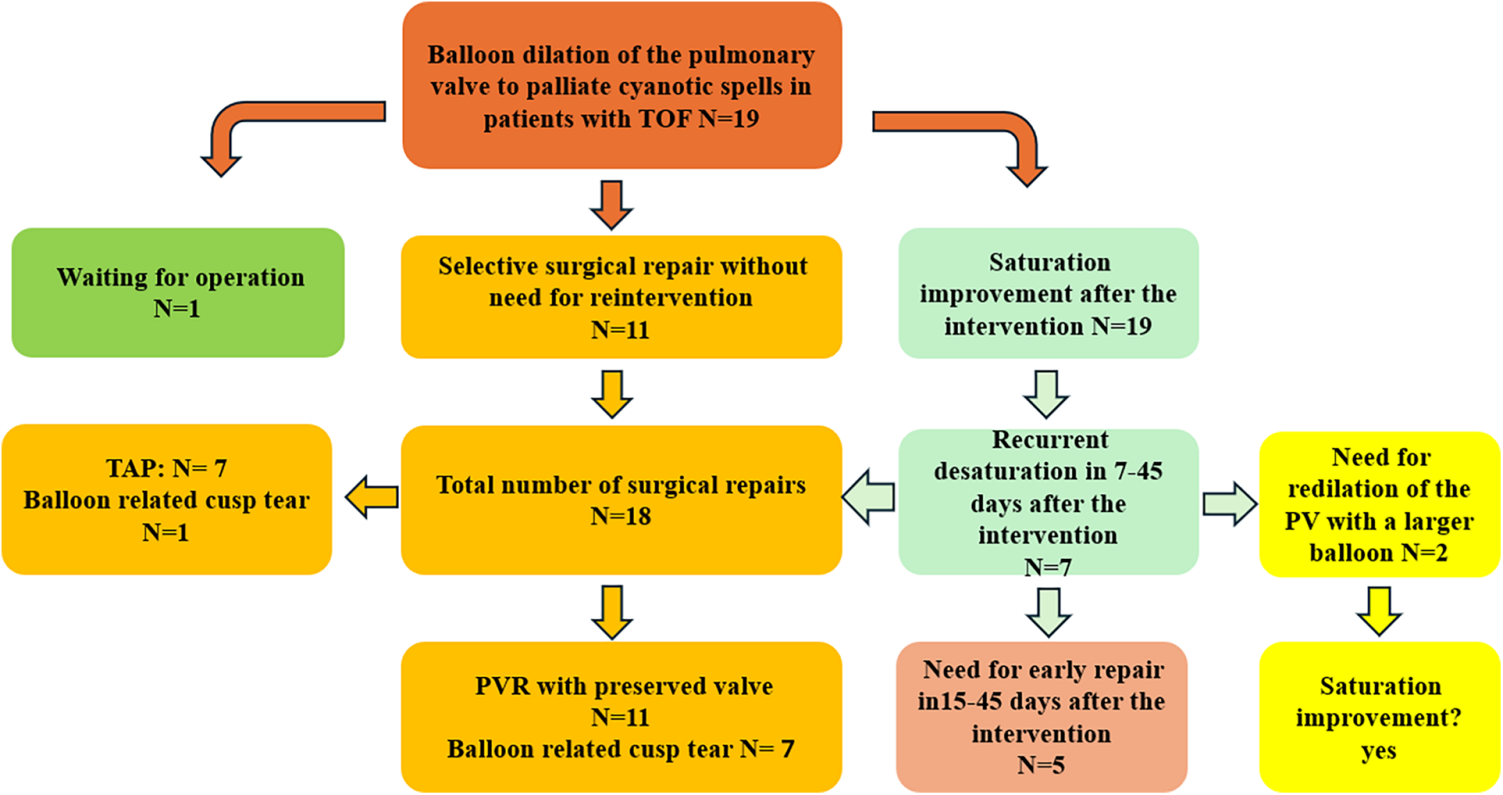
Patient flow chart diagram illustrating the patient cohort, including surgical strategies. TOF, tetralogy of Fallot; PVR, pulmonary valve reconstruction.

The intervention was performed as an emergency in 13 patients admitted to the intensive care unit (ICU) needing oxygen; 5 were in neonatological intensive care. The median weight, age, and BSA at the intervention time were 3.3 kg (1.8–6.5), 35 days (1–240), and 0.2 m^2^ (0.14–1.33), respectively. There was no intervention-related complication or death. No pericardial or pleural effusion was documented 24 and 48 h after the intervention (Figure 1).

### Oxygenation and echocardiography after the intervention

All patients showed significant saturation improvement after the intervention within the first week, with increased median saturation values from 70% (45–98) to 90.4 (74–98) (*p*-value = 0.03).

**Figure 2:**
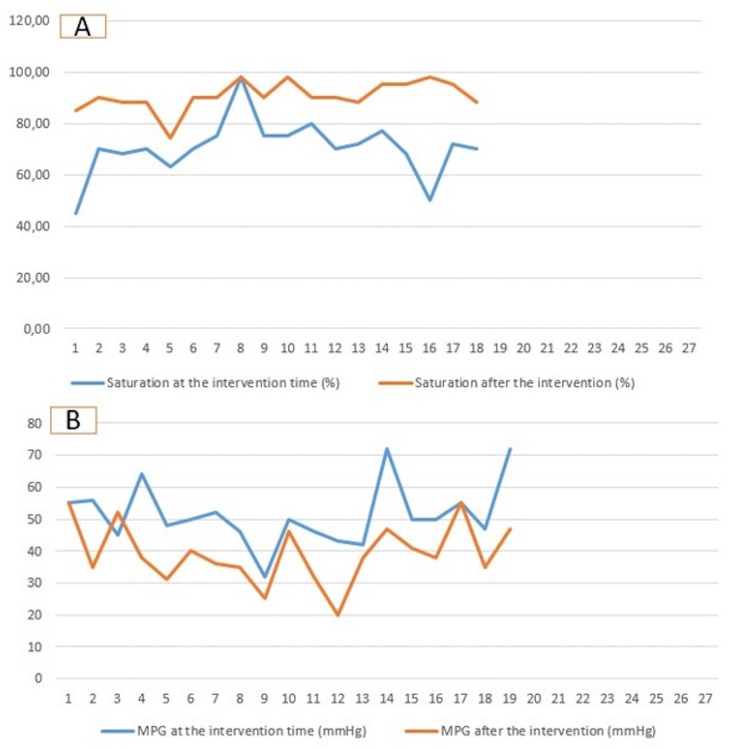
Diagram demonstrating the saturation **(A)** and the mean pressure gradient **(B)** at the time of intervention and at the time of surgical repair. MPG, mean pressure gradient.

The echocardiographic findings showed a reduction in the median value of the mean pressure gradient from 50 mmHg (32–72) to 38 mmHg (20–55) (*p*-value = 0.08) (Figure 2). Two patients documented severe pulmonary regurgitation, while the rest documented mild regurgitation. The recurrent desaturation within 7 45 days after the intervention occurred in 7 patients. The rest of the patients (*n* = 12) showed stable saturation (>88%) until the operation time.

### Need for reintervention to improve the saturation

Two patients needed pulmonary valve redilation by recurrent desaturation 7 and 12 days after the intervention. The first patient was 3.8 kg with cyanotic spells and recurrent seizures. The initial pulmonary valve size was 6.6 mm and was first dilated with an 8-mm low-pressure balloon (balloon/valve 1.2). One week later, the valve needed to be redilated with a 10 mm balloon (balloon/valve 1.4) by constantly decreasing saturation. The dilation was efficacious, with significant saturation improvement. No seizures were documented, and the patient was operated on 150 days after the intervention. In this patient, the decision for redilation was preferred to early repair due to incomplete neurologic diagnostic of the epilepsy at the time of the second intervention.

The second patient was premature [30 weeks gestational age (GA)] and weighed 1.8 kg. The pulmonary valve was 4.5 mm and was first dilated with a 6-mm balloon (balloon/valve 1.3). Two weeks later, the patient needed a valve redilation with an 8-mm balloon (balloon/valve 1.8) by recurrent desaturation. The surgical repair was performed with pulmonary valve reconstruction 210 days after the intervention.

### Need for early surgery

Five patients needed unplanned corrective surgery due to recurrence of desaturation 12, 15, 20, 30, and 45 days after the intervention. The patients’ weight at the operation time was 3, 3.3, 4.5, 4.7, and 5.8 kg, respectively. In these patients, the right ventricular outflow tract was narrow and had hypercontractility despite the increased dose of B-blocker and good hydration. Due to these patients’ well configured valve leaflets and annulus, early repair was superior to transcatheter stenting (to avoid losing the chance of surgical valve preservation). The pulmonary valve reconstruction (PVR) was amenable in 3 patients and transannular patching (TAP) in 2.

### Surgical findings and balloon-related leaflets tear

**Figure 3:**
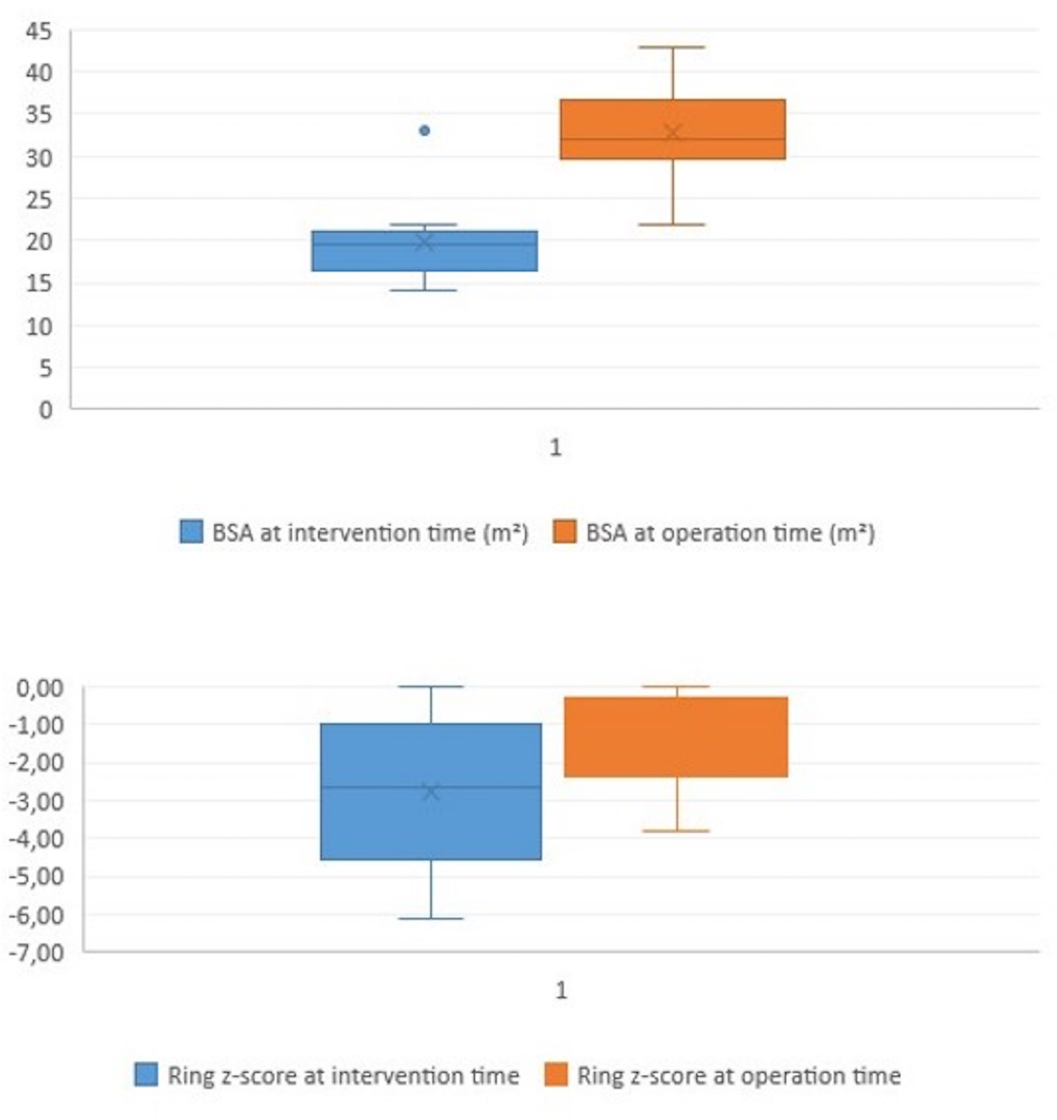
Boxplot diagram illustrating the BSA and the pulmonary ring z-score at the time of intervention (pre) and at the time of surgical repair (post).

**Figure 4:**
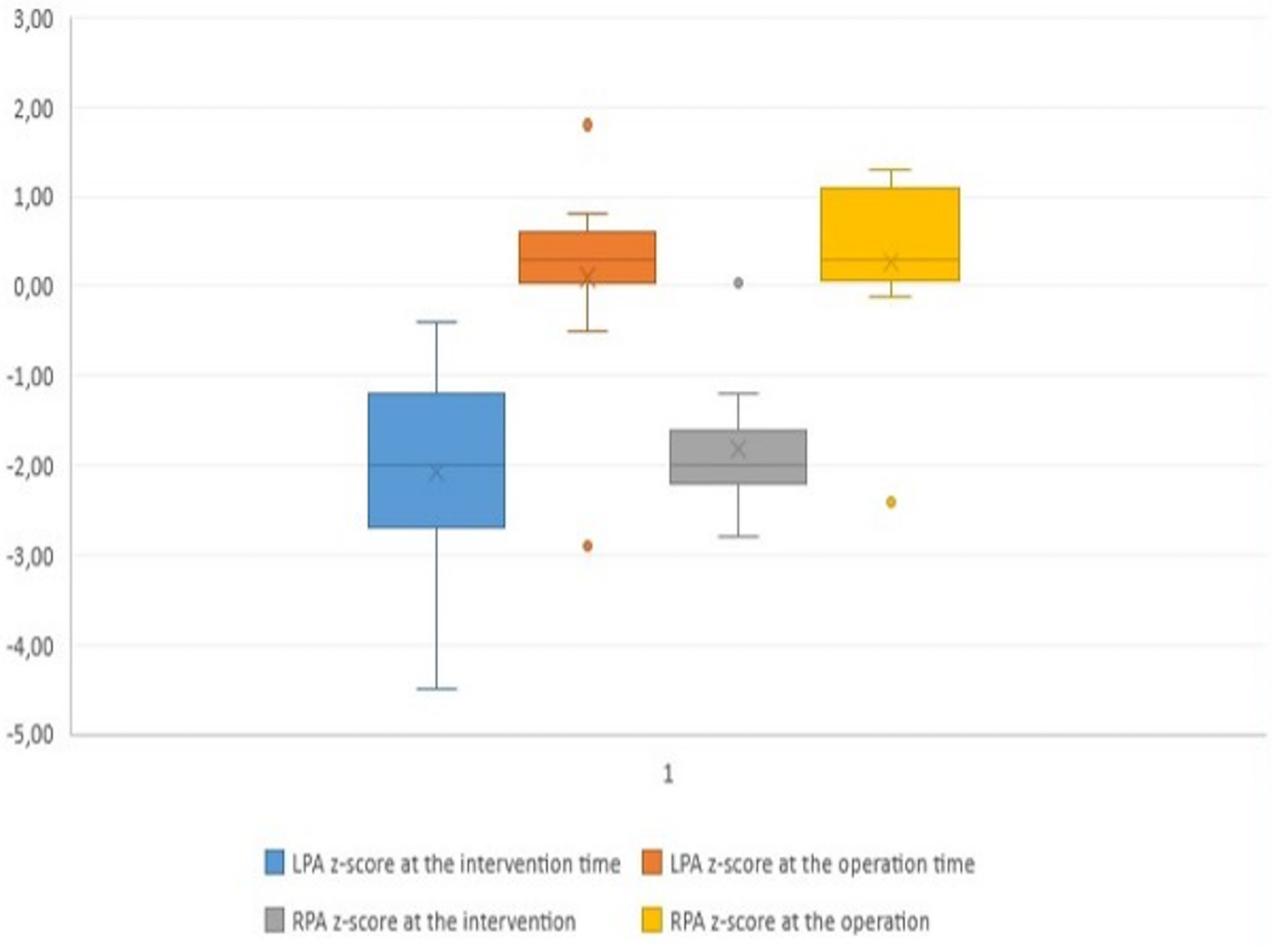
Boxplot diagram demonstrating the z-score of the pulmonary arteries at the time of transcatheter palliation (pre) and at the time of surgical repair (post). LPA, left pulmonary artery; RPA, right pulmonary artery.

The median pulmonary annulus z-score improved from −3.7 (−6.12 to −1.3) to −2.1 (−4.2 to −0.19) (*p*-value = 0.2) (Figure 3). The LPA z-score in the median value improved from −1.95 (−3.4 to −0.4) to 0.36 (−2.9 to 1.8) (*p*-value = 0.2), and the RPA z-score improved from −2 (−2.8 to 0.04) to 0.18 (−2.4 to 1.3) (*p*-value = 0.34) (Figure 4). Despite improving pulmonary growth, there was no significant relationship between the values at the times of the intervention and the operation.

The corrective repair was performed in 18 patients; 5 needed an early repair (as described in the previous paragraph), and 13 were operated on time. One patient was still waiting for the operation (250 days after the intervention; last weight was 6.7 kg). The median value of the weight, age, and BSA at the elective operation time was 6.5 kg (3.8–8.3), 180 days (46–525), and 0.32 m^2^ (0.22, 0.43).

Eight patients had mild to moderate tears of the pulmonary cusp. Five had no injuries, and the cusps in the rest were so thick and adherent that no injuries were documented.

In 11 patients, the pulmonary valve was reconstructed and preserved without needing transannular patching (TAP), while 7 required TAP due to a very dysplastic valve with thick adherent cusps. Out of 8 valves with documented BRCT 7 cusp tears could be repaired by sewing directly or by patch reconstruction in 1. No significant relation existed between balloon-related cusp injuries and the need for TAP (*p*-value = 0.13).

## Discussion

Although significant advancements have been made in the interventional and surgical techniques for palliating cyanotic patients with TOF, authors have not yet agreed on which palliative strategies are superior to those for symptomatic patients.

Neonates who have undergone early repair to palliate their cyanosis have an increased risk for reoperation, reintervention, and increased need for transannular patching. Those who have undergone early transcatheter dilation or stenting of the pulmonary stenosis have increased reinterventions. However, the outcomes of the 2 strategies seem similar regarding pulmonary growth and hemodynamics (7).

Both palliation with MBTS and transcatheter interventions have significantly improved the size of the pulmonary arteries (8, 9). However, antegrade pulmonary perfusion seems to provide more symmetrical pulmonary development than the pulmonary-systemic shunt, associated with higher mortality and morbidity rates than transcatheter palliation. While duct stenting can be effective in palliation, in cases of TOF, it may be ineffective due to the tortuous path of the duct, potentially leading to insufficient pulmonary perfusion and asymmetrical development (10, 11).

Several studies have been conducted over the last 4 decades to investigate the outcome of BVP in patients with TOF for palliating cyanosis (1–8, 12–14). However, the outcome of transcatheter dilation in symptomatic patients with TOF and dominant stenosis at the valve level still needs to be investigated due to the small number of patients needing transcatheter dilation. This study focuses on the outcome of the PVB as primary palliation and the impact of the BRCT on the surgical strategy regarding the need for TAP.

While previous studies described some intervention-related complications like cardiac tamponade, aortic regurgitation, infection and RVOT perforation (4–6, 13), we documented no intervention-related complications or deaths.

In our cohort, the saturation improved significantly in all patients in the first week after the intervention. However, 37% showed recurrent cyanotic spells or desaturation 7–45 days after the intervention, requiring a redilation or early repair. Concerns have been raised regarding cyanotic “spells” associated with balloon valvuloplasty, leading to questioning of its efficacy. While balloon valvuloplasty does not prevent these spells, it may even trigger them. By relieving an obstruction distal to the infundibulum, the procedure can lower systolic pressure and reduce the systolic wall stress that opposes ventricular contraction into the right ventricular outflow tract. This reduction may lead to closure and result in what is referred to as an “infundibular spasm,” similar to what has been observed following valvuloplasty for isolated pulmonary stenosis (2, 15). This phenomenon likely explains the transient increase in cyanosis and the failure of palliation in the patients who required early surgery or redilation.

In patients who needed redilation (10.5%), either the pulmonary anulus size was underestimated, or the size of the balloon used for primary dilation was small and inadequate to improve pulmonary flow. We had to choose the size of the balloon for the second intervention to be 2 mm larger than the first to achieve a satisfactory result. In patients who needed early repair (26%), the increased narrowing observed in the right ventricular outflow tract after the intervention despite the B-blocker was the cause of the recurrent desaturation. In this case, RVOT stenting or early repair was required to treat the symptoms. In our center, early repair is superior to RVOT stenting in that it increases the chance of preserving the pulmonary valve when the patients are fit for the operation. The surgical repair was successfully delayed in 74% of patients (including those who needed redilation), and the saturation was satisfactory and constant with propranolol. In our cohort, TAP was needed in 38% of the patients, compared with 29% (3) and 43% (12) of patients mentioned in previous studies published in 2016 and 1998.

Although the z-scores for the pulmonary ring and pulmonary arteries improved at the time of surgery compared to those at the time of intervention, the differential improvement was not statistically significant. Nevertheless, the sizes of the pulmonary arteries at surgery remained within normal limits, with zscore median values of 0.36 for the RPA and 0.18 for the LPA. Furthermore, the growth was symmetrical and sufficient, enabling the surgical procedure to be carried out without any need for reconstruction of the pulmonary artery. The only statistically significant documented improvement in our cohort was in oxygen saturation. This observation agrees with two previous studies (2, 4).

Our cohort's documented balloon-related cusp tears were intraoperatively mild to moderate in severity and amenable to being directly sewn or constructed. The presence of the BRCT did not impact the decision on the surgical strategy; moreover, 87% of valves with BRCT were preserved without needing TAP.

## Limitations

As this was a retrospective observational study, potentially crucial secondary outcome measures could not be fully assessed. Additionally, the single-center data collection limited the number of patients recruited and reflects the ambitions and experiences of only a small number of interventionalists.

## Conclusion

In the cases of tetralogy of Fallot involves pulmonary valve stenosis, the transcatheter dilation of the pulmonary stenosis is safe and could be effective in some patients for palliating cyanosis and improving the saturation and development of the pulmonary arteries. It can allows the surgery to be performed at the optimal time in some patients. The cusp injuries and tears caused by the balloon during the intervention differ in severity and have no negative impact on the surgery's technical decision. Based on our results, most cusp tears can be constructed or directly sewn, and the corresponding valve can, in most cases, be preserved when the pulmonary annulus is well developed.

## Data Availability

The original contributions presented in the study are included in the article/Supplementary Material, further inquiries can be directed to the corresponding author.
